# EEG Emotion Recognition Using AttGraph: A Multi-Dimensional Attention-Based Dynamic Graph Convolutional Network

**DOI:** 10.3390/brainsci15060615

**Published:** 2025-06-07

**Authors:** Shuai Zhang, Chengxi Chu, Xin Zhang, Xiu Zhang

**Affiliations:** 1 Tianjin Key Laboratory of Wireless Mobile Communications and Power Transmission, Tianjin Normal University, Tianjin 300387, China; 2310310032@stu.tjnu.edu.cn (S.Z.); ecezhang@tjnu.edu.cn (X.Z.); 2 Faculty of Computer Science and Information Technology, Universiti Malaya, Kuala Lumpur 50603, Malaysia; 22102131@siswa.um.edu.my

**Keywords:** electroencephalography, emotion recognition, feature matrix, attention mechanism, graph neural network

## Abstract

Background/Objectives: Electroencephalogram (EEG) signals, which reflect brain activity, are widely used in emotion recognition. However, the variety of EEG features presents significant challenges in identifying key features, reducing redundancy, and simplifying the computational process. Methods: To address these challenges, this paper proposes a multi-dimensional attention-based dynamic graph convolutional neural network (AttGraph) model. The model delves into the impact of different EEG features on emotion recognition by evaluating their sensitivity to emotional changes, providing richer and more accurate feature information. Results: Through the dynamic weighting of EEG features via a multi-dimensional attention convolution layer, the AttGraph method is able to precisely detect emotional changes and automatically choose the most discriminative features for emotion recognition tasks. This approach significantly improves the model’s recognition accuracy and robustness. Finally, subject-independent and subject-dependent experiments were conducted on two public datasets. Conclusions: Through comparisons and analyses with existing methods, the proposed AttGraph method demonstrated outstanding performances in emotion recognition tasks, with stronger generalization ability and adaptability.

## 1. Introduction

Emotion recognition plays a crucial role in human–computer interaction (HCI) [1,2], as it enables machines to understand, perceive, and adapt to human emotional and psychological states, thus exhibiting more empathetic characteristics during interactions. Achieving this capability is of great significance for enhancing the intelligence of human–computer interaction. Emotion, as a complex mental function, involves multiple aspects, such as affective, cognitive, behavioral, and physiological responses. Emotions are not only closely related to our emotional experiences but also influence cognitive processing, decision-making, and social interactions. Understanding and recognizing emotions, particularly in the context of HCI, is of great significance for enhancing the intelligence of systems and improving the user experience.

The technical approaches to emotion recognition are mainly divided into two categories: methods based on non-physiological signals and methods based on physiological signals [3]. The former decodes emotional states through external representations, such as facial expressions, body posture, or voice signals [4,5], while the latter recognizes emotions by capturing changes in physiological signals, such as electroencephalography (EEG), electromyography (EMG), and electrocardiography (ECG), that reflect internal states [6,7]. EEG, as a signal directly recorded from the cortical brain, has the unique advantage of reflecting emotion-related neural activity.

Emotion consists of multiple components: physiological responses (e.g., heart rate, breathing), emotional experiences (e.g., joy, anger, fear), and behavioral responses (e.g., facial expressions, speech intonation). These components are interwoven and collectively form our emotional experience. The mechanisms of emotion involve the coordinated action of multiple brain regions, such as the limbic system (including the amygdala), prefrontal cortex, and insula. These regions work together to generate different emotional responses through the evaluation of external stimuli and the regulation of internal states.

The methods for emotion description are primarily categorized into two approaches: the discrete basic emotion description and the dimensional approach. The discrete method classifies emotions into several basic states, such as joy, sadness, and surprise [8], while the dimensional approach quantifies emotions through continuous dimensions, like valence and arousal [9]. Recent hybrid models have emerged to integrate the advantages of both approaches [10]. Meanwhile, in EEG emotion recognition, common feature extraction methods can be categorized into time-domain and frequency-domain methods. Time-domain features, such as Hjorth parameters and fractal dimension feature, capture the temporal dynamics of the signal, while frequency-domain features extract frequency-related characteristics by decomposing the EEG signal into multiple frequency bands (such as delta, theta, alpha, beta, and gamma) [11].

EEG-based emotion recognition not only has significant technical potential but also has profound implications for human emotional understanding and mental health. Emotions are a vital component of human behavior and cognition, and EEG signals, as a direct reflection of brain activity, provide a real-time, non-invasive method for monitoring emotional states. By accurately identifying emotional changes, EEG emotion recognition technology helps deepen the understanding of emotional regulation mechanisms, providing critical biological insights for affective neuroscience research [12].

In clinical applications, EEG emotion recognition can be used in the mental health field, particularly in the diagnosis and treatment of emotional disorders, such as depression and anxiety, offering an objective tool for emotional assessment [13]. For instance, EEG signals can help identify early signs of emotional dysregulation, providing data support for personalized treatment plans. Additionally, in adaptive systems (such as smart environments and personalized therapy) and HCI, EEG emotion recognition allows systems to perceive users’ emotional states in real-time, optimizing interaction experiences and enhancing the intelligence and adaptability of the systems.

Therefore, EEG emotion recognition not only advances the research in affective neuroscience but also holds broad practical application prospects, especially in areas such as psychological diagnostics, clinical intervention, and human–computer interaction [14].

EEG signals can reflect the brain’s electrical activity, and different brain wave frequencies are closely related to emotional states. For example, alpha waves are typically associated with a relaxed state; beta waves with high activation states, such as tension and anxiety; and delta waves with deep sleep and relaxation. Evoked potentials are electrophysiological responses of the brain to external stimuli, and changes in emotional states can affect the characteristics of evoked potentials, with responses being more pronounced in emotional activation states. Event-related potentials (ERPs) reflect the brain’s response to specific events, and emotional stimuli often lead to significant changes in the ERP waveform, especially in the expression of components such as the P300 wave [15], which reflects the impact of emotions on the brain’s information processing. By combining multimodal EEG signals for analysis, researchers can more comprehensively explore the brain’s response under different emotional states, revealing the neurophysiological mechanisms of emotion [16]. These studies not only provide theoretical support for emotion recognition technologies but also offer potential biomarkers for the diagnosis and treatment of emotional disorders.

This paper proposes a novel EEG emotion recognition model—multi-dimensional attention-based dynamic graph convolutional neural network (AttGraph). This model focuses on evaluating the relative importance of different EEG features through a multi-dimensional attention convolution module and a global attention module, dynamically selecting the most discriminative features. This not only reduces redundant features but also significantly improves the computational efficiency of the model. At the same time, the AttGraph model explores the role of various EEG features in emotion recognition tasks. The in-depth exploration and precise selection of EEG features not only enhance the accuracy and robustness of emotion recognition but also make the model more adaptable and capable of cross-subject emotion recognition.

Specifically, the contributions of this work and the proposed AttGraph method are as follows:

(1) Propose the Multi-dimensional Attention Dynamic Graph Convolutional Neural Network

The AttGraph models EEG signals by combining multi-dimensional convolution layers, fully exploring the latent information of different dimensional features. Unlike traditional graph convolution networks, the AttGraph better captures the multi-dimensional complexity of EEG signals by modeling the EEG features across multiple dimensions. This approach enhances the model’s ability to recognize emotional states, strengthens its robustness and flexibility, and effectively addresses the complexity of emotional changes and subject-to-subject variability.

(2) Investigated the Impact of Various EEG Signal Features on Emotion Recognition

The AttGraph model deeply explores the role of different EEG signal features in emotion recognition tasks. By analyzing various EEG features individually, the model can evaluate each feature’s sensitivity to emotional state changes. This comprehensive study of EEG features fills the gap in previous emotion recognition tasks where feature diversity was insufficiently considered, providing richer and more precise feature information for emotion recognition.

(3) Combination of the Attention Mechanism and Graph Convolution

The AttGraph model integrates the attention mechanism with graph convolution, dynamically selecting and weighting EEG features to improve the efficiency of feature selection. The attention mechanism helps the model focus on key features that are highly related to emotional changes among the vast range of EEG features, effectively reducing the impact of redundant features. Through this method, the model not only simplifies the computational process but also demonstrates stronger discriminative ability under different emotional states, thereby improving the accuracy of emotion recognition.

## 2. Related Work

In this section, we will introduce some basic knowledge of EEG features and graph neural networks, which form the foundation of the AttGraph model.

### 2.1. EEG Signal Features

Various EEG features play a crucial role in emotion recognition, as they can capture subtle changes in brain activity during emotional states from different dimensions, such as time-domain, frequency-domain, and distributional features [17]. Different EEG features, such as differential entropy features, power spectral density features, and asymmetric features, provide richer and more precise information, helping to better understand the impact of emotional changes on brain activity. This paper explores the influence of various EEG signal features on emotion recognition, addressing the previous lack of attention to the diversity of EEG features in emotion recognition research.

The following is a detailed introduction to five common EEG features:

(1) Differential Entropy (DE) Feature

Differential entropy (DE) is a feature used to quantify the complexity and uncertainty of EEG signals. It measures the irregularity of the signal by calculating its probability distribution, reflecting the degree of chaos in brain activity. The differential entropy feature captures small changes in the EEG signal, which are often closely related to fluctuations in emotional states and the nervous system’s response. Studies have shown that different emotional states lead to varying degrees of complexity in the cerebral cortex, making differential entropy commonly used in emotion recognition tasks [18].

(2) Power Spectral Density (PSD) Feature

The power spectral density (PSD) is typically used to represent the power distribution of the signal across different frequency ranges. PSD reflects the strength of electrical activity in the brain across various frequency bands, making it significant for emotion recognition. It should be noted that band-specific features (e.g., alpha and theta waves) are calculated by integrating the PSD over corresponding frequency bands. In fact, band-specific features essentially represent subsets of PSD within specific frequency ranges, with the two maintaining a mathematically derived relationship.

(3) Differential Asymmetry (DASM) Feature

Differential asymmetry (DASM) reflects the asymmetry in the activity of the cerebral cortex between the two hemispheres of the brain in certain frequency bands. The activity of the left and right hemispheres plays an important role in emotional regulation. Many studies have shown that negative emotions (such as anxiety and fear) are often associated with increased activity in the right hemisphere, while positive emotions (such as happiness and relaxation) are linked to increased activity in the left hemisphere. The DASM feature calculates the activity differences between the left and right sides of the brain, reflecting the asymmetry of the brain during emotional states [19].

(4) Reasonable Asymmetry (RASM) Feature

Reasonable asymmetry (RASM) is an improvement upon the differential asymmetry feature that is aimed at further enhancing emotion recognition accuracy. RASM considers the relative activity between the two hemispheres of the brain rather than just the absolute power difference. By introducing specific normalization and standardization methods, it eliminates asymmetry errors that may be caused by individual differences or external factors, such as noise or movement [18]. RASM not only helps identify the left–right brain activity differences during emotional states but also better captures brain region activity patterns related to emotional changes. Compared with DASM, RASM provides more stable and accurate emotion recognition results, especially in complex emotional states.

(5) Differential Causality (DCAU) Feature

Differential causality (DCAU) is a feature extracted through analyzing the causal relationships between EEG signals. Causality analysis methods can reveal the information flow and interaction patterns between different brain regions. In emotion recognition, the collaborative activity of different brain regions is an important mechanism for emotional regulation, so causality analysis can effectively reflect the brain’s activity patterns during emotional states. During emotional states, the activity of certain brain regions may influence the activity of other regions. Therefore, by analyzing the DCAU feature, the dynamic brain network during emotional regulation can be reflected. DCAU features are particularly valuable for emotion recognition, as they capture the brain network coordination and information flow during emotional changes.

### 2.2. Graph Convolutional Neural Network

The graph convolutional neural network (GCNN) was proposed by Kipf and Welling as an extension of traditional convolutional neural networks and was designed to handle data with irregular structures. A GCNN demonstrates significant advantages when processing graph-structured data, particularly in capturing spatial and topological structures [20,21]. A GCNN can effectively describe the relationships between different channels, improving emotion recognition performance. EEG signals themselves have significant spatiotemporal characteristics, and by using graph neural networks, emotion-related patterns and features can be more effectively extracted based on the graph structure.

Wang et al. established a typical GCNN model on an EEG-derived graph, which integrates intra-frequency functional connectivity graphs and cross-frequency functional connectivity graphs [22]. Experimental results show that a GCNN outperforms a CNN in functional connectivity graph representations. While a GCNN can capture the relationships between different channels by constructing an adjacency matrix, most methods rely on a fixed adjacency matrix definition, which is typically based on prior knowledge or the spatial topology of the electrodes. Although this approach can reflect the spatial characteristics of EEG signals to some extent, it does not consider the dynamic changes in brain activity across different emotional states. With the continued development of deep learning, particularly the rise of graph neural networks, researchers have begun to realize that the adjacency matrix can be dynamically adjusted through adaptive learning. Song et al. proposed a Dynamical Graph Convolutional Neural Network (DGCNN) model, which can simultaneously extract discriminative features and functional connectivity information [23]. Unlike traditional GCNNs, the DGCNN dynamically learns the intrinsic relationships between different channels through training, as represented by an adjacency matrix. Although the DGCNN dynamically adjusts the graph structure during training to focus on changes in the graph, it lacks a dynamic feature selection and weighting mechanism for EEG features, which may lead to a more complex computational process and lower accuracy when dealing with a large number of EEG features.

Compared with traditional methods, the AttGraph model in this paper combines the attention mechanism with graph convolution, enabling automatic selection and the weighting of key features that are highly correlated with emotional changes, reducing the impact of redundant features, and effectively improving computational efficiency. This innovative approach not only captures the important features in emotional signals more effectively but also significantly enhances the accuracy of emotion recognition. The DGCNN lacks this fine dynamic adjustment in feature selection, which may lead to a more complex computational process and lower accuracy when handling large amounts of EEG features. In contrast, the AttGraph demonstrates stronger discriminative power across different emotional states, significantly improving the accuracy and efficiency of emotion recognition.

## 3. Methodology

### 3.1. AttGraph for EEG Emotion Recognition

**Figure 1:**
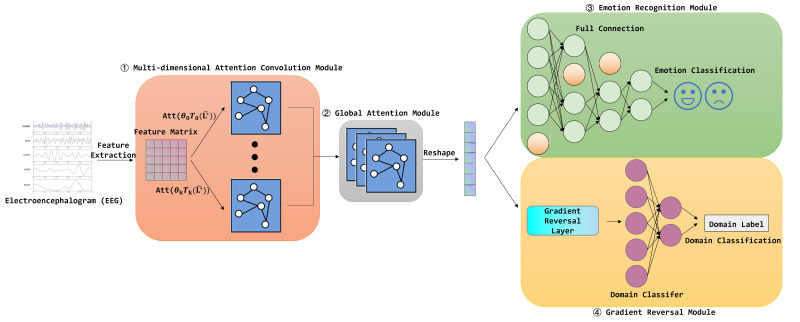
The framework of the proposed AttGraph method. The AttGraph method dynamically selects critical EEG features through multi-dimensional attentional convolution module, enhances discriminative patterns via global attention module, and incorporates a gradient reversal layer to mitigate inter-subject variability.

The AttGraph model proposed in this paper aims to perform emotion recognition using EEG signals. Its structural design effectively reduces the redundant features and enhances the efficiency of feature selection, thereby better exploring and utilizing the dynamic changes and complex patterns of EEG signals. The framework is shown in Figure 1.

Specifically, the input to the AttGraph model comes from EEG features extracted from multiple frequency bands, with each EEG channel treated as a node in the model. Next, the AttGraph dynamically selects and weights the EEG features in the multi-dimensional attention convolutional layer to enhance the efficiency of feature selection. Each convolutional layer generates a feature matrix dynamically through a custom attention mechanism, allowing it to adaptively highlight key channels and frequency bands related to emotional changes. This layer helps the model focus on the key features that are highly correlated with emotional changes from a large number of EEG features, and these key features are refined across multiple graph convolution layers, effectively reducing the impact of redundant features.

Next, the model further employs a global attention module to assign attention weights to different channels, enabling the model to focus on the features most relevant to emotional changes and strengthen the key parts of the emotional signals. By dynamically weighting and fusing the results of different attention convolution layers, the model further enhances the expressiveness of the emotional features, ensuring the effective transmission of key signal features, thus improving the overall emotion recognition performance. Finally, through a multi-layer fully connected network and a softmax classification layer, the AttGraph model is able to accurately predict the emotional category labels of the input EEG features.

Finally, to improve the domain generalization, the model introduces a gradient reversal layer (GRL), which adjusts the features during the backpropagation process to reduce the impact of differences in EEG signals between different subjects. Additionally, an extra domain classifier is used for domain discrimination, helping the model achieve domain adaptation and improving emotion recognition across subjects. The AttGraph model can simultaneously output both emotion prediction results and domain prediction results, achieving high-precision emotion classification and cross-subject generalization capabilities.

The design of the AttGraph model fully considers the complexity and dynamic changes of emotional EEG signals. Through its innovative mechanism for automatically selecting and adjusting key features, the model can effectively capture and adapt to EEG changes under different emotional states, further optimizing emotion recognition results through adaptive learning. Overall, the AttGraph model not only improves the accuracy of emotion recognition but also demonstrates strong applicability and robustness across subjects, making it widely applicable to various emotion recognition tasks.

### 3.2. Algorithm for AttGraph

To further elaborate on the proposed AttGraph model, this section will progressively introduce the algorithmic process of the AttGraph model, from input processing to emotion classification and domain adaptation implementation.

First, the input data x represents various feature representations of EEG signals, such as the differential entropy, power spectral density, and asymmetry features. The data is organized as a three-dimensional tensor with the shape (*B*, *E*, *F*), where B represents the batch size, E represents the number of electrodes, and F represents the number of frequency bands corresponding to each electrode. These input features serve as the node features for the graph neural network and are fed into the model for processing.

Next, the AttGraph uses multi-layer attention convolutions to extract features from the input. Unlike traditional graph convolution methods, which rely on static adjacency matrices, the AttGraph entirely adopts a data-driven attention mechanism to learn the relationships between nodes. In each graph convolution layer, the model first applies a linear transformation to map the input features into a latent space:

(1) H=XW

Here, $X\in R^{B\times E\times F}$ represents the input EEG feature tensor, $W\in R^{F\times F^{\prime }}$ is the learnable projection matrix, and $F^{\prime }$ is the output feature dimension of each layer in the multi-layer attention convolution. $H\in R^{B\times E\times F^{\prime }}$ represents the node representations in the latent space.

Subsequently, the model constructs a dynamic adjacency matrix to represent the strength of connections between electrodes:

(2) $$A_{att}=softmax(HAH^{T},dim=-1)$$

Here, $A\in R^{F^{\prime }\times F^{\prime }}$ is the trainable attention weight kernel, and $A_{att}\in R^{B\times E\times E}$ is the symmetric attention graph, which quantifies the mutual relationships between electrodes. The softmax operation is applied to the electrode dimensions (i.e., the 2nd and 3rd dimensions), normalizing the connections between electrodes for each sample. This operation ensures that the relative connection strength between electrodes is properly weighted.

To ensure symmetry, the adjacency matrix is symmetrized by the following operation:

(3) $$A_{att}=\frac{A_{att}+{A_{att}}^{T}}{2}$$

This symmetrization ensures that the relationships between electrodes are bidirectional and consistent, which aids information propagation in the graph convolution.

The model then performs a weighted aggregation of the features using this adjacency matrix to complete one layer of graph convolution:

(4) $$Z=A_{att}H$$

The computational complexity of each graph convolution layer is $O(B\times E^{2}\times F^{\prime })$.

Multiple attention convolution layers are stacked to achieve deeper emotion-feature-modeling capabilities. The model further utilizes a global attention module to perform weighted aggregation of the outputs from all electrode nodes, calculating the attention score $\beta_{i}$ for each node and performing weighted fusion of the corresponding feature $z_{i}$:

(5) $${Z^{\prime }}_{i}=\beta_{i}\cdot Z_{i}$$

Here, $\beta_{i}=\frac{exp(\omega_{i})}{\sum_{j=1}^{E}exp(\omega_{j})}$, where $\omega_{i}$ is a scalar score computed for node i and measures the importance of node to the final emotion recognition result. $Z_{i}$ represents the feature vector of node i.

The final fused features are then flattened and passed through a four-layer fully connected network to complete the emotion classification:

(6) $$\hat{y}=Softmax(W_{4}\sigma (W_{3}\sigma (W_{2}\sigma (W_{1}Flatten(Z^{\prime })))))$$

Here, $W_{i}$ is the weight matrix of the fully connected layers, which connect different layers of the model. The features are processed after passing through the linear transformation at each layer.

To enhance the model’s generalization ability in cross-subject emotion recognition tasks, the AttGraph introduces a gradient reversal layer for domain adaptation. The intermediate features $Z^{\prime }$ are fed into this module:

(7) $$Z_{rev}=GRL(Z^{\prime },\alpha )$$

Its gradient is multiplied by −α during backpropagation to maximally confuse the distribution of the source domain and target domain, allowing the model to learn domain-invariant feature representations. Subsequently, these reversed features are fed into the domain classifier. By minimizing the emotion classification loss and maximizing the domain classification loss through an adversarial objective, the model ultimately enhances the cross-subject emotion recognition accuracy.

In summary, the AttGraph model, with its attention-driven graph convolution structure at its core, integrates feature selection mechanisms and domain adaptation strategies to achieve the deep modeling of EEG emotional information and cross-subject generalization, demonstrating superior performance in complex emotion recognition tasks.

## 4. Experiments

In this section, we will conduct extensive experiments on two commonly used emotional EEG datasets, SEED [8] and SEED-IV [24], to evaluate the performance of the proposed AttGraph method.

### 4.1. Emotional EEG Datasets

The SEED dataset is provided by the Brain Computing and Intelligence Laboratory at Shanghai Jiao Tong University for emotional recognition research. In the experiment, 15 subjects (7 males and 8 females, average age 23.27 years) watched 15 movie clips from six Chinese films (including positive, neutral, and negative emotions). Each clip lasted about 4 min, with a 5 s prompt at the beginning and a 45 s self-assessment period at the end. After watching each clip, the subjects filled out an emotional response questionnaire.

SEED-IV is an extension of the SEED dataset, where each subject watched six movie clips in four emotional categories (happy, sad, fear, neutral). Data was recorded using a 62-channel ESI NeuroScan system and an SMI eye tracker. Each clip lasted about 2 min, with a 5 s prompt and a 45 s self-assessment period. The participants’ ratings were based on their actual feelings while watching the clips rather than what they thought the movie clips should be like.

### 4.2. EEG Emotion Recognition Experiment on the SEED Dataset

(1) Subject-Independent Experiment on the SEED Dataset

We investigated five common EEG features to evaluate the proposed emotion recognition method: differential entropy (DE), power spectral density (PSD), differential asymmetry (DASM), rational asymmetry (RASM), and differential caudality (DCAU). These features were extracted from five frequency bands $\left(\delta ,\theta ,\alpha ,\beta ,\gamma \right)$. To extract the EEG features, the signals were first downsampled to a 200 Hz sampling rate, and the records contaminated by electromyography (EMG) and electrooculography (EOG) artifacts were manually removed. The EOG signals were used to identify blink artifacts, and a bandpass filter between 0.3 and 50 Hz was applied to remove noise. The EEG data for each movie clip were divided into 1 s non-overlapping segments, and features were extracted from each segment. The number of EEG features extracted from each frequency band and a summary of the different feature types can be found in Table 1. The data input to the model has a dimensionality of (64, 62, 5). In this way, we ensure that the multi-dimensional characteristics of emotional EEG signals are comprehensively captured, providing rich information for emotion recognition.

To ensure the reproducibility of the model, we used the following hyperparameter configuration in the experiments: a batch size of 64 and training epochs 40 during the training phase, with a learning rate of 0.01. The batch size in the testing phase was the same as in the training phase. To guarantee the reproducibility of the results, we set the random seed (seed = 100) and used GPU acceleration for the computation. The optimizer used was Adam, and CrossEntropyLoss was employed as the loss function. The domain adaptation was implemented using a GRL, with the α parameter set to 0.1, which controls the strength of domain adaptation during training. Specifically, the domain classifier was implemented using a fully connected neural network with 100 hidden units, batch normalization, ReLU activations, and a LogSoftmax output. We also conducted experiments with different values of the α parameter and found that selecting other values resulted in a decrease in both the accuracy and standard deviation by approximately 2–3%. All experiments were conducted based on these fixed hyperparameter settings, ensuring that the reported classification accuracy is reliable and can be reproduced under the same configuration.

In the subject-independent experiment, we used the Leave One Subject Out (LOSO) cross-validation method to evaluate the performance of the AttGraph model in the EEG emotion recognition. Specifically, in the LOSO cross-validation, the EEG data from 14 subjects were used to train the model, while the EEG data from the remaining 1 subject were used as the test data. The experiment was repeated to ensure that the EEG data from each subject was used as the test data once. Each epoch took approximately 17 s, and the total number of model parameters was about 77,000. We then calculated the average classification accuracy and standard deviation for the five EEG features, with the results shown in Table 2.

As shown in Table 2, there is a significant difference in the contribution of different EEG features to emotion recognition in the AttGraph model. The differential entropy feature was the most effective at capturing the complexity and uncertainty changes in the brain activity, and thus, achieved the highest recognition accuracy. The power spectral density features also performed well, as they effectively captured emotional changes in the frequency domain. In contrast, differential asymmetry and reasonable asymmetry features performed poorly, possibly due to the dynamic nature of emotional states and the strong influence of individual differences. The differential caudality features provided unique insights into the brain region coordination and information flow. Although their accuracies were lower than the first two, they still had certain value. These results suggest that the AttGraph model could flexibly utilize different EEG features, dynamically weighting and selecting key features from EEG signals to highlight those with strong discriminative power in emotion state changes, thereby significantly improving the accuracy and robustness of emotion recognition.

**Figure 2:**
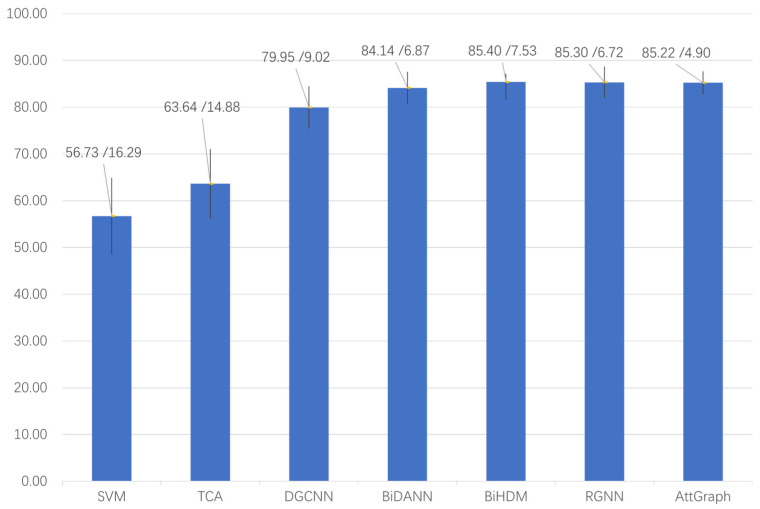
Comparison of the accuracy and standard deviation of the SVM [25], TCA [8], DGCNN [23], BiDANN [26], BiHDM [27], RGNN [28], and AttGraph in the subject-independent experiment on the SEED dataset.

Furthermore, to further demonstrate the performance of the AttGraph model, we compared it with several popular EEG emotion recognition models. The comparison results are shown in Figure 2.

The AttGraph model performed excellently in the EEG emotion recognition tasks, showing significant improvements compared with traditional methods such as SVM and DGCNN. The accuracy of the SVM was 56.73% with a standard deviation of 16.29%, far below other methods, indicating considerable limitations in the traditional approaches for cross-subject emotion recognition tasks. The TCA achieved an accuracy of 63.64%, with a standard deviation of 14.88%, showing improvement over the SVM but still significantly lower than the AttGraph. Although the DGCNN achieved an accuracy of 79.95%, with a standard deviation of 9.02%, demonstrating the advantages of graph convolution, it still fell short of the AttGraph. In contrast, the AttGraph achieved an accuracy of 85.22%, with a standard deviation of 4.90%, and thus, outperformed both the SVM and DGCNN and offered higher stability. Compared with current state-of-the-art models, the AttGraph’s performance was also very close. For instance, BiDANN and BiHDM achieved accuracies of 85.4% and 85.3%, with standard deviations of 7.53% and 6.72%, respectively, while the RGNN had an accuracy of 84.14% and a standard deviation of 6.87%. These results show that the AttGraph was competitive with the most advanced deep learning methods in terms of both accuracy and stability, demonstrating its strong competitiveness and application potential in emotion recognition tasks.

To comprehensively evaluate the contribution of each module in the AttGraph model, we designed ablation experiments by sequentially removing key modules and comparing their performance. The experimental results are shown in Table 3. First, to verify the contribution of the multi-dimensional attention convolution module, we replaced it with a traditional GCN. The results showed that after removing this module, the model’s accuracy significantly decreased (from 85.22% to 81.75%) and the standard deviation increased. This indicates that the multi-dimensional attention convolution module played a crucial role in capturing and weighting key features across different channels, and thus, significantly improved the model’s accuracy and robustness. Specifically, in the context of emotion EEG signal processing, it effectively captured the correlations between channels, which enhanced the model’s stability.

Next, we replaced the global attention module with a simple direct summation method. The results show a slight decrease in accuracy (from 85.22% to 83.06%) after removing this module, but the impact was relatively small, indicating that the global attention module played a positive role in optimizing the feature fusion. It helped the model retain more critical feature information, which improved the accuracy and stability of the emotion recognition.

Finally, to validate the effect of the gradient reversal module, we removed this module. The experimental results indicate a slight decrease in the average accuracy (from 85.22% to 83.41%) and a slight increase in the standard deviation. This suggests that although the gradient reversal module was essential for cross-subject generalization, the model could still maintain a good performance even without it, especially when working with a relatively limited subject dataset. In particular, the model still performed well at the emotion recognition with the standardized data.

Through these ablation experiments, we not only verified the effectiveness of each module at the emotion EEG signal recognition but also gained a deeper understanding of the advantages and potential of the AttGraph model. The multi-dimensional attention convolution module played a central role in capturing and weighting the key channel features, and thus, significantly improved the model’s accuracy and robustness. The global attention module optimized the feature fusion, which helped the model retain more important information, although its contribution was not as critical as the multi-dimensional attention convolution module. The gradient reversal module enhanced the model’s generalization ability through cross-subject domain adaptation. Despite its relatively small impact when removed, it still helped to reduce the subject differences. These experimental results show that each module in the AttGraph model played an important role in the emotion EEG signal recognition, which further validated the model’s effectiveness and applicability.

(2) Subject-Dependent Experiment on SEED Dataset

**Figure 3:**
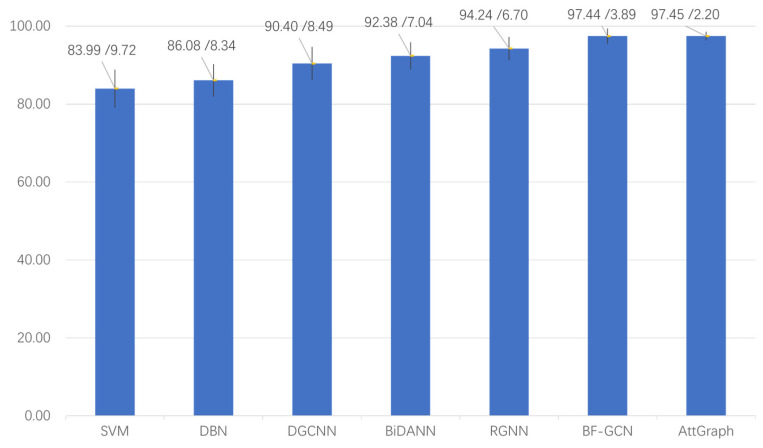
Comparison of the accuracy of the SVM [25], DBN [8], DGCNN [23], BiDANN [26], RGNN [28], BF-GCN [29], and AttGraph in the subject-dependent experiment on the SEED dataset.

In the subject-dependent experiment, each subject’s 15 EEG data recordings from a single session were used, with the first 9 as the training set and the remaining 6 as the test set. As in the subject-independent experiment, we compared the AttGraph model with several popular EEG emotion recognition models. The comparison results are shown in Figure 3.

As can be seen from the figure, the AttGraph model performed excellently in the EEG emotion recognition tasks. Compared with traditional methods, such as the SVM and DBN, the AttGraph significantly improved the accuracy, where it reached 97.45%, a notable increase compared with the SVM (83.99%) and DBN (86.08%). At the same time, the AttGraph also outperformed models like the DGCNN (90.4%) and BiDANN (92.38%), demonstrating the powerful potential of combining graph convolution and attention mechanisms in emotion recognition. Compared with current state-of-the-art models, like the RGNN (94.24%) and BF-GCN (97.44%), the AttGraph performed remarkably well, with a lower standard deviation (2.20%), showcasing the higher stability and generalization ability. Therefore, the AttGraph demonstrated strong competitiveness in emotion recognition tasks, where it outperformed traditional methods and was on par with the most advanced technologies.

### 4.3. EEG Emotion Recognition Experiments on the SEED-IV Dataset

(1) Subject-Independent Experiment on the SEED-IV Dataset

In this experiment, we explored two common EEG features to evaluate the proposed emotion recognition method: differential entropy (DE) and power spectral density (PSD). The features were extracted from five frequency bands $\left(\delta ,\theta ,\alpha ,\beta ,\gamma \right)$. The number of EEG features extracted from each frequency band and the summary of different feature types are shown in Table 4. Similar to the SEED dataset, the data input into the model had a dimensionality of (64, 62, 5).

In the subject-independent experiment, we still used the LOSO method to evaluate the performance of the AttGraph model in EEG emotion recognition, with an approximate runtime of 9 s per epoch, and calculated the average classification accuracy and standard deviation for the two EEG features. The results are shown in Table 5.

The experimental results of the AttGraph model on the SEED-IV dataset show that the differential entropy feature could effectively capture the complexity and uncertainty in EEG signals and was somewhat related to the changes in emotional states. Although this feature performed well in the emotion classification, its classification accuracy was lower than that on the SEED dataset due to the more complex fluctuations of emotional states. The PSD feature, while reflecting the brain’s electrical activity intensity across different frequency bands and capturing the frequency components in emotional changes, showed a relatively low accuracy in the four-class task. This may have been because in the multi-class emotional state, the discriminative power of the PSD feature was weak, and it could not effectively distinguish the subtle differences between the different emotional states. Next, we compared the AttGraph model with the same models used in the subject-independent experiment of the SEED dataset, and the results are shown in Table 6.

The experimental results show that the AttGraph model performed exceptionally well in the EEG emotion recognition tasks. Compared with traditional methods, such as the SVM (accuracy of 37.99%) and TCA (accuracy of 56.56%), the AttGraph demonstrated a significant performance improvement, where it achieved an accuracy of 78.36% with a standard deviation of 9.61%, indicating a high accuracy and stability in cross-subject emotion recognition. Compared with methods like the DGCNN (accuracy of 52.82%), the AttGraph further enhanced the recognition performance, and also performed exceptionally well among the state-of-the-art methods, surpassing the BiDANN (accuracy of 65.59%), BiHDM (accuracy of 69.03%), and RGNN (accuracy of 73.84%). These results demonstrate that the AttGraph had strong competitiveness in the emotion recognition tasks, where it effectively overcame the limitations of traditional methods and achieved results that were on par with or surpassed the existing cutting-edge technologies.

**Figure 4:**
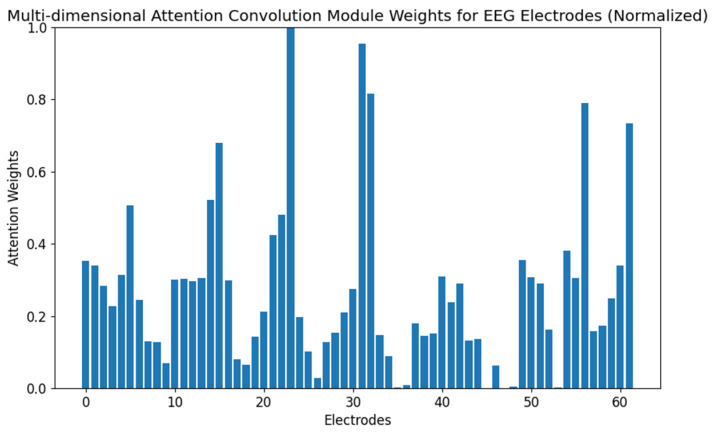
The distribution of weights in the multi-dimensional attention convolution module in the subject-independent experiments on the SEED-IV dataset.

**Figure 5:**
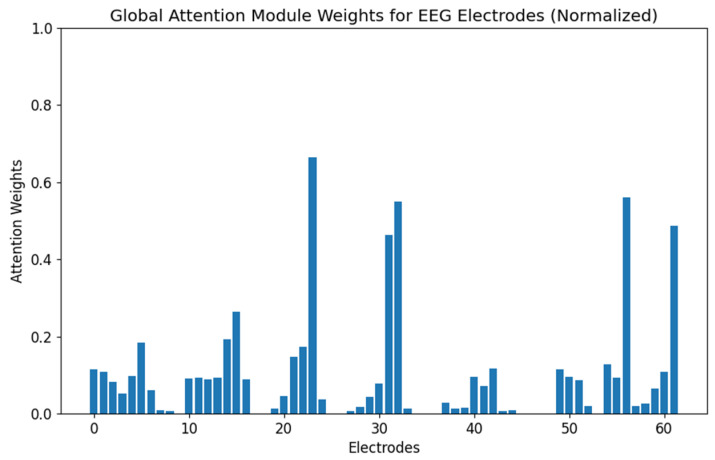
The distribution of weights in the global attention module in the subject-independent experiments on the SEED-IV dataset.

To better understand the behavior of the AttGraph model in emotion recognition tasks, we conducted attention visualization experiments. By visualizing the attention weights of each node in the graph convolution network, we analyzed the model’s focus on different electrode nodes under various emotional states. Figure 4 and Figure 5 show the importance weights of EEG electrode nodes as processed by the multi-dimensional attention convolution module and the global attention module during the emotion classification task, respectively.

As shown in Figure 4, the electrodes that exhibited higher attention weights (15, 23, 25, 31, 32, 33, and 50) spatially aligned with the emotion-related cortical areas identified in prior neuroimaging studies [24], particularly in the prefrontal and temporal regions known to be involved in affective processing. This indicates that the AttGraph could automatically identify key emotional channels, demonstrating its excellent feature learning and spatial perception abilities. Compared with Figure 4, the weight distribution in Figure 5 is sparser, suggesting that the model was able to focus its attention on the most relevant electrode channels during the decision stage, and thus, removed redundant information.

**Figure 6:**
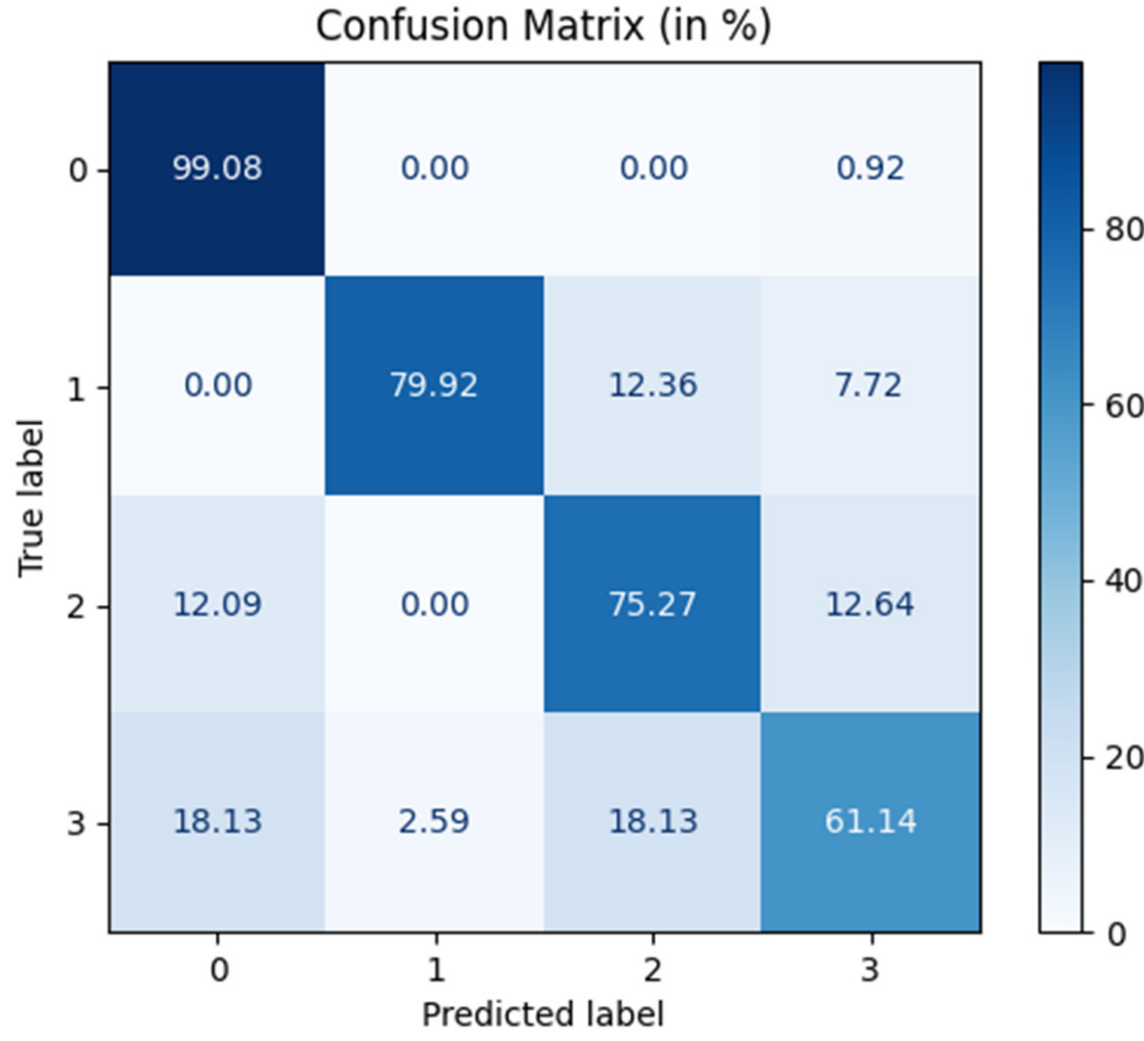
Confusion matrix of subject-independent experiments in the SEED-IV dataset.

Finally, Figure 6 presents the confusion matrix for the AttGraph model in the multi-class emotion recognition task, showing the model’s classification accuracy and confusion for the four emotional states: neutral (0), sadness (1), fear (2), and happiness (3).

From a physiological perspective, the high recognition rate of neutral emotions (99.08%) may be related to their stable EEG characteristics with minimal emotional fluctuation, making them easier to distinguish. The low recognition rate of happiness (61.14%) was associated with the complexity of its EEG signals, which involved dynamic activation across multiple brain regions (such as the reward system and prefrontal cortex), and significant individual differences, which made it difficult to accurately distinguish the signals. In contrast, the higher recognition rates for sadness and fear (79.92% and 75.27%, respectively) were closely related to the key role of the amygdala, which plays a central role in these negative emotions, which caused the EEG signals to exhibit clear and stable characteristics in the lower frequency bands (such as θ and α waves), which facilitated model recognition. Although sadness and fear share some similarities, their physiological responses differ, which allowed the model to effectively differentiate between these two emotions. Therefore, the AttGraph model aligned well with these physiological behaviors and effectively captured and distinguished EEG patterns associated with different emotional states.

(2) Subject-Dependent Experiment on the SEED-IV Dataset

In this experiment, we used the same experimental method and the same EEG emotion recognition models as in the subject-dependent experiment on the SEED dataset. The experimental results are shown in Table 7.

In the subject-dependent experiments, the AttGraph model demonstrated an outstanding performance, where it significantly surpassed the traditional methods, such as the SVM, DBN, and DGCNN. Specifically, the accuracy of the SVM was 56.61%, with a standard deviation of 20.05%, which was much lower than the other methods, indicating significant limitations of traditional approaches in emotion recognition tasks. The DBN achieved an accuracy of 66.77%, with a standard deviation of 7.38%, showing an improvement over the SVM, but it was still significantly lower than the AttGraph. The DGCNN achieved an accuracy of 69.88%, with a standard deviation of 16.24%, and despite using graph convolution methods, it still could not outperform the AttGraph. In contrast, the AttGraph achieved an accuracy of 93.92%, with a standard deviation of 2.78%, where it performed not only better than the SVM, DBN, and DGCNN but also with a higher stability. Compared with the current state-of-the-art models, the AttGraph also performed exceptionally well. For example, the BF-GCN achieved an accuracy of 89.55%, with a standard deviation of 10.95%, while the RGNN achieved an accuracy of 79.37%, with a standard deviation of 10.54%. These results demonstrate that the AttGraph exhibited strong competitiveness in emotion recognition tasks, particularly in the subject-dependent experiments, where its excellent performance and stability proved the model’s efficiency and potential for application.

To further evaluate the contribution of each module in the AttGraph model to the emotion recognition performance, we conducted an ablation study based on the subject-dependent experiments using the SEED-IV dataset. The experiment involved progressively removing key modules from the model and observing the changes in accuracy to analyze their impact on the overall performance of the model. The experimental results are shown in Table 8 below.

In the ablation study, we progressively removed key modules from the AttGraph model and observed their impacts on the performance. When the multi-dimensional attention convolution module was removed and replaced with a traditional GCN, the model accuracy dropped to 88.71%, with a standard deviation of 4.61%, indicating that the multi-dimensional attention convolution module is crucial for capturing complex emotional features in EEG signals. After removing the global attention module, the accuracy decreased to 90.68%, with a standard deviation of 3.10%, but the performance still exceeded that of removing the multi-dimensional attention module, suggesting that the global attention module helps focus on the key features of emotional changes. When the gradient reversal module was removed, the accuracy dropped to 91.21%, with a standard deviation of 6.81%. Although the accuracy decreased, the model still maintained good performance, indicating that this module plays a significant role in domain adaptation. The complete AttGraph model achieved an accuracy of 93.92%, with a standard deviation of 2.78%, showing the best performance and stability. Overall, the multi-dimensional attention convolution module and global attention module were critical for improving the model accuracy, while the gradient reversal module contributed to enhancing the model’s domain adaptation capability.

## 5. Discussions

The AttGraph model has made significant progress in EEG emotion recognition tasks and has deeply explored the impact of different EEG signal features on emotion recognition. First, the AttGraph model systematically evaluated the sensitivity of each feature to emotional changes through the individual analysis of multiple EEG features. This exploration fills the gap in traditional emotion recognition methods that insufficiently focus on feature diversity, providing more comprehensive and accurate feature information for emotion recognition. Second, the model cleverly combines attention mechanisms with graph convolution, dynamically selecting and weighting EEG features to focus on key features highly correlated with emotional changes, while reducing the interference from redundant information, thus enhancing the discriminative power in emotion recognition tasks. The AttGraph achieves adaptive focusing on key brain region electrodes through its multi-dimensional attentional convolution module and global attention mechanism, which aligns with previously reported neural mechanisms of emotion processing. Finally, our method outperformed the others in terms of the overall performance on both datasets, demonstrating a strong generalization ability.

At the same time, the AttGraph model performed excellently in the EEG emotion recognition tasks. In the SEED dataset, it significantly outperformed the traditional methods, such as SVM and DGCNN. Compared with the current state-of-the-art models, AttGraph’s performance was comparable to the BiDANN and BiHDM, and in some experiments, it even surpassed the RGNN. For the SEED-IV dataset, particularly in cross-subject emotion recognition, the AttGraph’s accuracy far exceeded that of the SVM and TCA, and it also outperformed the DGCNN and BiDANN. It should be noted that although the SEED and SEED-IV datasets have certain limitations in terms of participant age distribution (18–25 years) and cultural homogeneity. It is also important to consider that developmental and individual differences in the EEG asymmetry, particularly in frontal lobe activity and emotional processing, may influence emotion recognition results [30]. Moreover, the current results have demonstrated the significant application value of AttGraph.

## 6. Prospects for Future Research

Although the AttGraph model has achieved encouraging results in emotion recognition, there are still many potential improvements and extensions worth exploring. While the AttGraph model performed well on the single-subject data, improving cross-subject emotion recognition accuracy remains a challenge. This can be addressed by enhancing the domain adaptation techniques or leveraging more domain knowledge to improve the model’s generalization ability. Additionally, the explainability of emotion recognition models remains an important topic. Future work could further explore how to interpret the model’s decision-making process when making emotion classifications by combining the features of attention mechanisms and graph convolution, thereby enhancing the model’s transparency and reliability. Relevant research directions can refer to interpretable graph neural network (GNN) models, such as the prototype-based interpretable GNN proposed by Ragno et al. [31], and the interpretable spatio-temporal GNN using information bottleneck proposed by Fang et al. [32].

Regarding future directions, we plan to further explore the deployment of the AttGraph model in real-world applications, particularly by integrating it with BCI for real-time emotion recognition. Specific future work will focus on the following areas: (1) designing online adaptation protocols for clinical settings to address challenges arising from individual differences and environmental changes; (2) optimizing the model’s latency performance to meet real-time emotion-monitoring needs; (3) integrating the model with neurofeedback systems to promote its clinical applications in areas such as emotional regulation and anxiety reduction.

These concrete research directions not only aim to further improve the model’s usability and accuracy but also push forward the application of emotion recognition technology in the field of brain–computer interfaces, thus enhancing its potential in clinical monitoring and interventions for mental health.

## Figures and Tables

**Table 1:** The numbers of five EEG features extracted from each frequency band in the SEED dataset.

EEG Features	Band δ	Band *θ*	Band *α*	Band *β*	Band *γ*
DE	62	62	62	62	62
PSD	62	62	62	62	62
DASM	27	27	27	27	27
RASM	27	27	27	27	27
DCAU	23	23	23	23	23

**Table 2:** Average accuracy and standard deviation (%) of different features in subject-independent EEG emotion recognition experiments on the SEED dataset.

Model	Feature	Average Accuracy ± Standard Deviation (%)
AttGraph	DE	85.22 ± 4.90
	PSD	80.99 ± 5.21
	DASM	52.51 ± 12.39
	RASM	55.24 ± 11.46
	DCAU	75.29 ± 6.00

**Table 3:** Ablation study results of subject-independent experiments on the SEED dataset (%).

Model	Average Accuracy ± Standard Deviation (%)
Without multi-dimensional attention convolution module (replaced by GCN)	81.75 ± 6.12
Without global attention module (replaced by direct summation)	83.06 ± 5.19
Without the GRL	83.41 ± 5.09
AttGraph	85.22 ± 4.90

**Table 4:** Number of EEG features extracted from each frequency band in the SEED-IV dataset for the two EEG features.

EEG Features	Band δ	Band *θ*	Band *α*	Band *β*	Band *γ*
DE	62	62	62	62	62
PSD	62	62	62	62	62

**Table 5:** Average accuracy and standard deviation (%) of different features in the subject-independent EEG emotion recognition experiments on the SEED-IV dataset.

Model	Feature	Average Accuracy ± Standard Deviation (%)
AttGraph	DE	78.36 ± 9.61
	PSD	62.97 ± 9.42

**Table 6:** Compares the accuracy and standard deviation of the SVM, TCA, DGCNN, BiDANN, BiHDM, RGNN, and AttGraph in the subject-independent experiments on the SEED-IV dataset.

Model	Average Accuracy ± Standard Deviation (%)
SVM [25]	37.99 ± 12.52
TCA [8]	56.56 ± 13.77
DGCNN [23]	52.82 ± 9.23
BiDANN [26]	65.59 ± 10.39
BiHDM [27]	69.03 ± 8.66
RGNN [28]	73.84 ± 8.02
AttGraph	78.36 ± 9.61

**Table 7:** Comparison of the SVM, DBN, DGCNN, BiDANN, RGNN, BF-GCN, and AttGraph on the accuracy and standard deviation in the subject-dependent experiment on the SEED-IV dataset.

Model	Average Accuracy ± Standard Deviation (%)
SVM [25]	56.61 ± 20.05
DBN [8]	66.77 ± 7.38
DGCNN [23]	69.88 ± 16.24
BiDANN [26]	70.29 ± 12.63
RGNN [28]	79.37 ± 10.54
BF-GCN [29]	89.55 ± 10.95
AttGraph	93.92 ± 2.78

**Table 8:** Ablation study results of subject-dependent experiments on the SEED-IV dataset (%).

Model	Average Accuracy ± Standard Deviation (%)
Without multi-dimensional attention convolution module (replaced by GCN)	88.71 ± 4.61
Without global attention module (replaced by direct summation)	90.68 ± 3.10
Without the GRL	91.21 ± 6.81
AttGraph	93.92 ± 2.78

## Data Availability

The data that support the findings of this study are available from the SEED repository: https://bcmi.sjtu.edu.cn/home/seed/seed.html (accessed on 31 May 2025).
